# Incidental Detection of Human Herpesvirus-6 in Cerebrospinal Fluid Analysis: To Treat or Not to Treat?

**DOI:** 10.7759/cureus.25629

**Published:** 2022-06-03

**Authors:** Abdurrahman F Kharbat, Mithra Lakshmi-Narasimhan, Smita Bhaskaran, Sumesh Parat

**Affiliations:** 1 Pediatrics, Texas Tech University Health Sciences Center, Amarillo, USA

**Keywords:** viral panel, viral cns infection, cns infection, neonatal infection, hhv-6 encephalitis, human herpesvirus-6 (hhv-6)

## Abstract

Human herpesvirus-6 (HHV-6) is a ubiquitous beta herpes virus which can result in meningitis/ meningoencephalitis in humans. FilmArray meningitis/encephalitis panel (BioFire Diagnostics, Salt Lake City, UT) is employed in medical centers for the establishment of a central nervous system (CNS) infection, and HHV-6 is often positive. However, establishing HHV-6 as a cause of meningitis or encephalitis is difficult at the present time, as a polymerase chain reaction (PCR) test alone does not establish the etiology of the CNS infection. HHV-6 can be transmitted in a Mendelian fashion by integrating into the human genome (ciHHV-6 or chromosomally integrated HHV-6). We present a case of a 34-week gestational age neonate in the neonatal intensive care unit (NICU) who tested positive for HHV-6 through a panel screening, presumably due to ciHHV-6 infection. Knowledge and understanding of this phenomenon is essential in preventing misdiagnosis of active HHV-6 infection and subsequent unnecessary antiviral treatment.

## Introduction

Human herpesvirus-6 (HHV-6) is a DNA virus that demonstrates near-universal infection in childhood, affecting more than 80% of children by two years of age [1-10]. With a varied range of infections, HHV-6 has been shown to induce an asymptomatic disease state in a majority of those affected, and infrequently cause febrile seizures, encephalitis, or meningitis in some [1]. Of its two variants, HHV-6A and HHV-6B, the latter results in roseola infantum or sixth disease, which is associated with 10 to 20% of febrile seizures in young children [1].

HHV-6 establishes a latent state in monocytes and macrophages and can reactivate in vulnerable populations such as the immunocompromised [1,6,10]. In those affected with neurological disease, HHV-6 may result in long-term neurological sequela and significant morbidity [10]. Of its notable characteristics, HHV-6 has been recently shown to infrequently integrate into the genome of those it affects and may be transmitted vertically from parent to neonate through the hereditary transmission of chromosomal DNA (ciHHV-6) [3,10]. An analysis conducted in Japan demonstrated that HHV-6 integration was present in 0.21% of the population under study, with another study citing a higher prevalence of 1 to 2% [2], highlighting a recently recognized congenital condition that presents a unique mode of transmission and particular need for establishing nuances in patient treatment [1,3]. In a study of 43 cases of congenital HHV-6 infection, it has been reported that 86% of congenital HHV-6 infections are due to ciHHV-6 and only 14% had transplacentally acquired infection [11].

Use of BioFire’s FilmArray panel (BioFire Diagnostics, Salt Lake City, UT), an FDA-approved PCR panel that detects 14 pathogens (including bacteria, fungi, and viruses) commonly implicated in cases of encephalitis, is widely being used by many hospitals, and HHV-6 has been a commonly detected pathogen [2]. However, without an ability to delineate ciHHV-6 status or viral load, concerns arise over the propensity to attribute a central nervous (CNS) system condition to HHV-6 when the panel is simply detecting ciHHV-6 DNA. Therefore, establishing HHV-6 as a cause of meningitis or encephalitis is difficult at the present time, as a polymerase chain reaction (PCR) test alone does not establish the etiology of the CNS infection [3,10,12].

## Case presentation

A late preterm female neonate was born at 34+5 weeks gestation to a 21-year-old G4P1 mother with a history of genital herpes simplex virus (HSV) infection prior to pregnancy and active genital HSV lesions during labor. Mother had a positive HSV-2 lesion culture. Newborn examination revealed a normal neonate, not in any obvious distress. Labs obtained on the baby at 24 hours of life were negative for HSV surface culture, HSV CSF culture, and CSF HSV DNA PCR. The BioFire meningitis panel indicated a positive result for HHV-6. Moreover, blood PCR on the neonate was also positive for HHV-6 (Table 1).

**Table 1:** Infectious Disease Laboratory Workup HSV = herpes simplex virus, CSF = cerebrospinal fluid, DNA = deoxyribonucleic acid, PCR = polymerase chain reaction

	Result
HSV Surface Culture	Negative
HSV CSF Culture	Negative
HSV CSF DNA PCR	Negative
BioFire Meningitis Panel At 24 Hours	Positive
BioFire Meningitis Panel At 8 Days	Positive

After consultation with the pediatric infectious disease specialist, she received IV Ganciclovir for seven days. Further investigation revealed that the patient was HHV-6 IgG positive and IgM negative, and the patient’s mother was also found to be IgG positive and IgM negative, as seen in Table 2.

**Table 2:** Antibodies to Human Herpesvirus 6 IgM = Immunoglobulin M, IgG = Immunoglobulin G

	IgM	IgG
Patient	Negative	Positive
Patient’s Mother	Negative	Positive

The BioFire meningitis panel was tested again at that time and was found to be positive for HHV-6 again. Therefore, the decision to continue IV Ganciclovir for a total of 21 days was made. White blood cell levels were serially trended, and she developed clinically significant neutropenia with an absolute neutrophil count of 800 cells/mm^3^ on day 10 of therapy, which is a known side effect of Ganciclovir therapy. A review of the literature elucidated to us that the likely culprit in the positive HHV-6 result could be a vertical transmission of ciHHV-6 DNA. This is reinforced by the fact that the patient was IgM negative, indicating that there was no acute infection, and the IgG antibodies were transferred transplacentally, providing further protection to the patient. As the positive HHV-6 was a serendipitous finding in an asymptomatic neonate undergoing evaluation for HSV and literature review showing the possibility of ciHHV-6, a decision was made to stop the medication after consultation with the pediatric infectious disease specialist. We did not do any confirmatory tests and observed the patient in neonatal ICU (NICU) as she was admitted with feeding problems of prematurity. Subsequently, her WBC counts increased to within normal limits. The baby remained asymptomatic and had an uneventful clinical course.

## Discussion

With the advent of new technologies like the FilmArray (BioFire) panel, there has been an uptick in rates of HHV-6 detection in standardized viral screening. The preponderance of cases are asymptomatic, are not associated with encephalitis, and are most likely due to ciHHV-6 [5]. Without an ability to delineate ciHHV-6 status or viral load, concerns arise over the propensity to attribute a CNS condition to HHV-6 when the panel is simply detecting ciHHV-6 DNA. A positive test has been shown to usually be due to either integration within vertically transferred chromosomes or due to subclinical reactivation of latent HHV-6 virus [5,9,10]. Additional diagnostic testing modalities such as viral load or HHV-6 DNA qPCR tests on CSF and blood may be needed to confirm clinical significance [5,9-11]. Chromosomal integration will lead to the presence of HHV-6 genome in all the cells of the body and this results typically in high viral loads in these subjects. This can be measured by quantitative PCR and an HHV-6 qPCR greater than 1 x 10^6^ copies/ml of whole blood is strongly suggestive of ciHHV-6. Qualitative PCR in hair follicles and nails can definitely confirm ciHHV-6.

Clinical laboratory analysis studies have demonstrated the propensity of misinterpreting positive ciHHV-6 tests and highlight the waste of resources, grave risk of misdiagnosis, and risks associated with unnecessary treatments [10,11]. Laboratory results can also further elucidate important information regarding the patient’s immune status and highlight avenues where HHV-6 analysis can yield clinically significant information. In patients who are found to be truly HHV-6 positive, different ranges of CSF/blood replication ratios may in fact illustrate the patient’s immune status [10]. Findings indicate a CSF/blood replication ratio <1 is detected in immunocompetent patients, while a CSF/blood replication ratio >1 is detected in immunocompromised patients [10]. Notably, these findings may represent reliable indicators of immune status even before radiologic evidence of infection could be established, as initial brain MRI was altered in two of seven truly HHV-6 positive and immunocompromised patients, but six of seven had a CSF/blood replication ratio >1 [10].

Physicians evaluating patients who are HHV-6 positive through FilmArray (BioFire) testing must be aware of the ciHHV-6 entity so that they do not overreact to a laboratory test without looking at the whole clinical picture. The decision to intervene on a positive test result should take into consideration the patient’s immune status, clinical condition, and laboratory findings to circumvent grave adverse effects that result from the use of antiviral agents.

## Conclusions

Providers need to realize that most congenital HHV-6 infections are due to ciHHV-6, and HHV-6 infection is generally asymptomatic amongst young infants. Our experience demonstrates a need for recommendations to be made to subject patients who test HHV-6 positive from a panel to a simple qPCR test on whole blood and HHV-6 viral load, which would allow physicians to establish ciHHV-6 status. Without a routine protocol to establish ciHHV-6 status in such patients, physicians are left without a true understanding of whether antiviral agents such as ganciclovir are the appropriate clinical course of action, which is especially problematic given that their inappropriate use could result in significant morbidity due to bone marrow suppression resulting in anemia and neutropenia.
